# The role of passive surveillance and citizen science in plant health

**DOI:** 10.1186/s43170-020-00016-5

**Published:** 2020-10-30

**Authors:** Nathan Brown, Ana Pérez-Sierra, Peter Crow, Stephen Parnell

**Affiliations:** 1 Woodland Heritage, P.O. Box 1331, Cheltenham, GL50 9AP UK; 2 Tree Health Diagnostics and Advisory Service, Forest Research, Alice Holt Lodge, Farnham, Surrey, GU10 4LH UK; 3 Observatree, Forest Research, Alice Holt Lodge, Farnham, Surrey, GU10 4LH UK; 4 School of Science Engineering and Environment, University of Salford, Salford, M5 4WT UK

**Keywords:** Surveillance, Citizen science, Unstructured data, Early warning, Tree health

## Abstract

The early detection of plant pests and diseases is vital to the success of any eradication or control programme, but the resources for surveillance are often limited. Plant health authorities can however make use of observations from individuals and stakeholder groups who are monitoring for signs of ill health. Volunteered data is most often discussed in relation to citizen science groups, however these groups are only part of a wider network of professional agents, land-users and owners who can all contribute to significantly increase surveillance efforts through “passive surveillance”. These ad-hoc reports represent chance observations by individuals who may not necessarily be looking for signs of pests and diseases when they are discovered. Passive surveillance contributes vital observations in support of national and international surveillance programs, detecting potentially unknown issues in the wider landscape, beyond points of entry and the plant trade. This review sets out to describe various forms of passive surveillance, identify analytical methods that can be applied to these “messy” unstructured data, and indicate how new programs can be established and maintained. Case studies discuss two tree health projects from Great Britain (TreeAlert and Observatree) to illustrate the challenges and successes of existing passive surveillance programmes. When analysing passive surveillance reports it is important to understand the observers’ probability to detect and report each plant health issue, which will vary depending on how distinctive the symptoms are and the experience of the observer. It is also vital to assess how representative the reports are and whether they occur more frequently in certain locations. Methods are increasingly available to predict species distributions from large datasets, but more work is needed to understand how these apply to rare events such as new introductions. One solution for general surveillance is to develop and maintain a network of tree health volunteers, but this requires a large investment in training, feedback and engagement to maintain motivation. There are already many working examples of passive surveillance programmes and the suite of options to interpret the resulting datasets is growing rapidly.

## Background

Surveillance for new and emerging pests and diseases in the wider environment represents a significant challenge for regulatory bodies in plant health (Parnell et al. 2017; Carvajal-Yepes et al. 2019). There are large and complex landscapes to cover and often significant uncertainty in maps of host species. This is especially true in natural, as well as urban environments, which can be more complex than agricultural systems. Detecting new epidemics is a needle in the haystack problem, and in many cases pests and diseases have been established for many years, and already reached high prevalence, before they are first detected (Siegert et al. 2014; Wylder et al. 2018). Additional observations from outside the official regulatory survey network, reported by land-users (such as land owners, land managers, professional agents and tenant farmers), citizen scientists and concerned members of the public, can play a vital role in pest and disease detection. These reports are often termed “passive surveillance” or “volunteered geographic information” and have the potential to greatly enhance detection. Passive surveillance occurs as and when an observer (a member of the public, land-user or member of a citizen science scheme), both notices something of concern and is aware of, and uses, the mechanisms available to notify authorities. The resulting data is unstructured and “messy” in nature (Dobson et al. 2020). Interpreting and using them to estimate the prevalence of a pest and inform biosecurity measures is thus challenging. The use of species population data collected by citizen scientists has been the focus of recent reviews across ecology and conservation (Dobson et al. 2020; Larson et al. 2020; Isaac and Pocock 2015; Pocock et al. 2017a; Dickinson et al. 2010; Crall et al. 2015). We build on these foundations to highlight the role of passive surveillance data in plant health. This review will identify current areas of research that maximise the potential use of such data, including: the variety of forms in which passive surveillance occurs, giving examples of current citizen science schemes in plant health; approaches that are available for analysing data; and considerations for developing future schemes.

In recent years plant, animal and human ecosystems across the globe have been threatened by an increased number of introductions of exotic pests and diseases (Boyd et al. 2013; Jactel et al. 2020). Within plant ecosystems most arrivals of novel pests and diseases are linked to the movement of living plant material, as well as, its products, and are accelerated by the global trade and the movement of people (Brasier 2008; Aukema et al. 2010; Freer-Smith and Webber 2015). In each country, plant health services and National Plant Protection Organisations (NPPOs) act to safeguard the biosecurity of plants whilst facilitating sustainable economic growth. Increasingly this balance is addressed by using a risk-based approach to ensure that effort is targeted and based on an assessment of the overall costs and benefits to society (Spence 2020; Spence et al. 2019a, b; Giovani et al. 2020). Government efforts to tackle pests and diseases involve pre-border, at the borders and inland activities. This means governments and regional organisations work internationally to reduce the likelihood of pests arriving, ensure checks are in place at borders to reduce the opportunities of pests arriving and establishing (Defra 2014, 2018). However, these processes are not infallible and cannot avert all outbreaks, so significant post border surveillance within agricultural, forest and natural environments are also required.

Post-border or inland surveillance activities are primarily carried out by NPPOs whose staff are trained to identify and sample for quarantine pests and diseases. Surveillance in this context is targeted to specific locations and hosts, following a structured design to provide a statistical basis and facilitate interpretation of the resulting data. Such surveys are best described as “active surveillance” (Hester and Cacho 2017). Surveillance can be designed to fulfil several objectives depending on the stage of a pest outbreak (Parnell et al. 2017). If a pest is not yet known to be present in a population, then detection surveys are conducted to find outbreaks in their early stages and most often used to confirm (with a prescribed level of certainty) the absence of the pest (Ciubotaru et al. 2018; Parnell et al. 2015). Here, surveys are usually risk-targeted to maximise the probability of pest detection (Bourhis et al. 2019; Hyatt-Twynam et al. 2017; Parnell et al. 2014; Martinetti and Soubeyrand 2019). If a pest is known to be present, then the focus of surveys changes to either delimitation of infested areas, or to estimates of the pests prevalence and spatial extent (EFSA 2019; Hauser et al. 2016; Brown et al. 2017a). These surveys generally rely on unbiased data from representative, rather than risk-targeted surveys. Following the implementation of a pest control intervention, prevalence estimation and mapping surveys may also be conducted to establish the effectiveness of the measures taken i.e. has there been a reduction in prevalence or the spatial extent of a pest population (Cunniffe et al. 2015, 2016). Finally, if an eradication attempt has occurred, detection surveys may once again be undertaken to determine success of interventions. Members of the public or land managers who report disease symptoms to the plant health authorities increase the likelihood of detection in surveillance programmes (Hester and Cacho 2017; Baker et al. 2018) and have a potential role across the full range of surveillance objectives. For example, the first detection of the oriental chestnut gall wasp (*Dryocosmus kuriphilus*) on sweet chestnuts in England was made by an amateur gall enthusiast who submitted samples to the official laboratory for identification (Morath et al. 2015).

Passive surveillance has been defined as “any encounter with a pest by members of the public that is reported to the relevant authority” (Hester and Cacho 2017). Increased access to websites and smartphone technology has greatly eased the ability of the public to engage with surveillance in recent years (Dobson et al. 2020; Pocock et al. 2017a; Dickinson et al. 2010). Although such reports, via phone, email and letter writing have long played an important role in the detection and diagnosis of tree pests and diseases (Gilbert et al. 2005; Potter et al. 2011). More recently an increasing number of tailored programmes have been used effectively to gather reports of rare events, such as new introductions and for low cost targeted surveillance (Pocock and Evans 2014; Meentemeyer et al. 2015; Hallett and Hallett 2018).

Passive surveillance programmes have been identified as an important pathway to engage with stakeholders and involve them directly in management efforts (Marzano et al. 2015). The success of prior initiatives and the importance of volunteered data to first detections has been recognised by policy makers. In the UK, a recent invasive species report identified the need to develop a “Citizen Army” to detect future threats and the crucial role for local volunteer groups to engage with detection and monitoring (Environmental Audit Committee 2019). These aspirations are dwarfed by action elsewhere, New Zealand’s Biosecurity 2025 programme aims to insure that 80% of the public will understand what they need to do to report a pest or disease if they find it (New Zealand government 2018).

Detections that occur through passive surveillance represent fortuitous contributions that depend on individuals both detecting an issue within the environment and deciding they should report the discovery to the authorities. These reports occur at times and places where it is convenient for the public to participate and, as such, are unstructured and potentially biased in distribution (Isaac and Pocock 2015; Baker et al. 2018; Johnston et al. 2020). This systematic error contravenes a fundamental assumption of most statistical approaches (that data is a representative random sample of the wider population) (Dobson et al. 2020; August et al. 2020). For analysts and policy makers there are clear limitations to how these data can be used, but there is no denying the practical usefulness of additional detections to plant health officials (Ryan et al. 2018). It remains an important challenge for researchers to develop methods for use with passive surveillance data, so that any bias can be understood and quantified (August et al. 2020; Boakes et al. 2016).

**Fig. 1:**
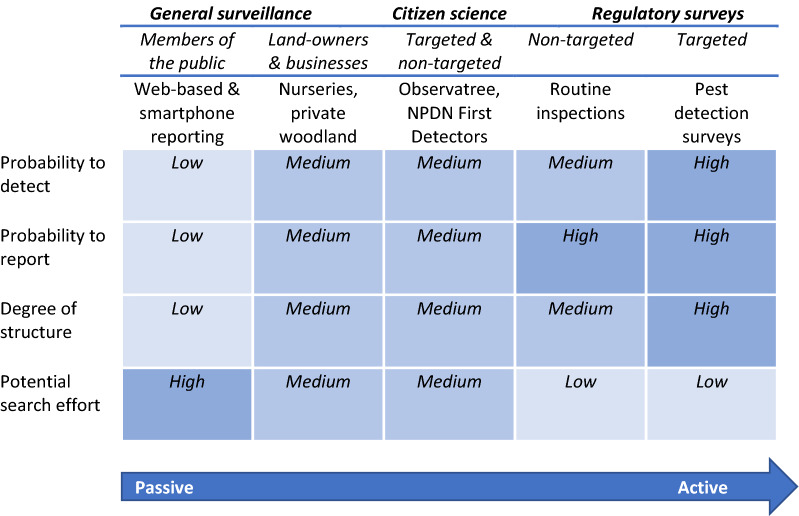
The ‘passive to active surveillance spectrum’ in plant health and suggested overall probability to detect, report, degree of structure in data collection, and potential search effort for different sources of surveillance. Figure developed from Hester and Cacho (2017)

Passive surveillance is rarely a purely passive process, with training materials and events often employed to increase the number of and accuracy of reports (Meentemeyer et al. 2015; Hallett and Hallett 2018; Crall et al. 2011). At this point reporting ceases to be purely opportunistic, with additional structures such as citizen science programmes favouring certain types of reporting behaviour (Boakes et al. 2016). In fact, most volunteer reporting methods currently available will contain both passive and active components (Hester and Cacho 2017), and individual programmes will present specific challenges to analyst’s dependent on the methods employed (Dobson et al. 2020). In this review we aim to characterise current approaches across the ‘passive to active surveillance spectrum’ (Hester and Cacho 2017) (Fig. 1) and highlight specific challenges that arise when working with datasets collected during passive surveillance. By understanding these challenges observer efforts can be best quantified and most reliably used to inform plant health officials, helping to reduce the burden on active surveys and improve pest and disease management outcomes. A final section of this review focuses on how the challenges of working with passive surveillance data can be mitigated, to a greater or lesser extent, through project design.

## The nature of passive surveillance

Within passive surveillance, Hester and Cacho (2017) distinguished different forms, general surveillance and citizen science. General surveillance represents one extreme of the passive surveillance continuum (Hester and Cacho 2017) here the discovery of a new pest or disease occurs purely by chance, when an individual (landowner, public) is not specifically looking to make a discovery. Citizen science (in its many forms) represents a more organized process with active promotion and training, which sits between general surveillance and active surveillance on the continuum (Fig. 1).

### Citizen science in surveillance

There are different terms (e.g. community science, crowd science, crowd-sourced science) and different understandings when referring to citizen science (Riesch and Potter 2014; Auerbach et al. 2019). Here we take a broad view of citizen science as the “involvement of volunteers in research” (Dickinson et al. 2010) and consider it a “tool” for undertaking research and monitoring, while also engaging with many people” (Pocock et al. 2014), however we make the distinction from general surveillance as citizen science programmes have specific hypotheses, goals or target species. Well-designed citizen science projects can usefully inform research, decision making and policy formation (Dickinson et al. 2010). Most citizen science programs that involve surveillance monitor numerous species over broad geographic regions, with the idea that data will ultimately be useful for a broad spectrum of questions. For example, the British Trust for Ornithology (Table 1) coordinates over 60,000 dedicated observers to collect biological data describing the distribution of over 100 bird species and has built a robust, long-term dataset that can document change (Harris et al. 2020). In plant health, many citizen science projects have effectively focused on finding new, rare or invasive organisms such as *Phytophthora ramorum*, the invasive plant pathogen responsible for sudden oak death (SOD) in California (Meentemeyer et al. 2015). Since 2008 a project in California and Oregon has run “SOD blitzes”, these events trained volunteers to identify *P. ramorum* symptoms and use a mobile mapping tool. Volunteers were then engaged to survey local forests and urban parks, with the resulting data improving the accuracy of predictive maps of the current distribution (Lione et al. 2017). Citizen science has also been used to monitor distribution, abundance, of pest species such as the horse-chestnut leaf-miner (*Cameraria ohridella*) in the UK (Pocock and Evans 2014).

**Table 1:** Additional information regarding projects discussed in this review

Project name	Region	Description	Website
Be a smart ash	Denver, Colorado, USA	Mapping the locations of urban ash (*Fraxinus* spp.) trees for early detection and planning for emerald ash borer arrival	https://beasmartash.org
Brigit	Great Britain	Determining the distribution of potential vectors for *X. fastidiosa*. Focusing on the spittle produced in spring by a wide range of froghoppers/spittlebugs and leafhoppers	www.spittlebugsurvey.co.uk
BTO breeding birds survey	United Kingdom	Monitoring the population changes of common and widespread breeding birds, producing population trends for 117 bird species. The structured survey involves visits to an allocated 1-km square, to count all the birds you see or hear while walking transects	www.bto.org/our-science/projects/bbs
Cape citizen science	Greater Cape Floristic Region, South Africa	Reporting locations and pictures of unhealthy plants with a focus on *Phytophthora cinnamomi*	https://citsci.co.za
Conker tree science	Great Britain	Surveys of horse chestnut *Aesculus hippocastanum* focusing on horse chestnut leaf miner and its natural enemies. Surveys were conducted in 2010 and 2020	www.conkertreescience.org.uk
Covid symptom study	United Kingdom	A smart phone application that allows users to regularly report their health status to track Covid 19 infections in the community	https://covid.joinzoe.com
First detector	United States of America	First detectors are tree health volunteers who help with early detection and receive training through online materials and in person workshops	www.firstdetector.org
National Biodiversity Network (NBN)	United Kingdom	The NBN Atlas is an online tool that provides a platform to link to multiple sources of information about UK species and habitats. It aims to facilitate learning about and understanding the UK’s wildlife	https://nbnatlas.org
National Plant Diagnostic Network (NPDN)	United States of America	A network of 70 diagnostic laboratories across the USA who diagnose plant pests and pathogens	https://www.npdn.org
Non-native species portal (GB‐NNSIP)	Great Britain	Tools and information to support the no-native species strategy including links for reporting sightings	https://www.nonnativespecies.org
Observatree	Great Britain	Tree health volunteers, trained to identify and survey for 22 priority pests and diseases (more details in case study 2)	www.observatree.org.uk
OPAL tree health	Great Britain	Reporting the presence of pests and diseases on Oak (*Quercus robur* and *Q. petraea*), Ash (*Fraxinus excelsior*) and Horse Chestnut trees (with a focus on six of the most serious pest and disease threats, “The Most Unwanted”). Project active between 2013 and 2019	www.imperial.ac.uk/opal/surveys/treehealthsurvey
TreeAlert	Great Britain	An online portal for the reporting and diagnosis of all tree pests and diseases in Great Britain (more information in case study 1)	https://treealert.forestresearch.gov.uk
Tree bodyguards	Europe	Students from 10 European countries installed thousands of fake clay caterpillars in trees to detect traces of teeth, beaks or mandible left by caterpillar predators. Their observations inform studies investigating the effects of climate on tree defences and defoliator predation	https://www6.inrae.fr/tree-bodyguards

Within this review we consider passive surveillance both in terms of general surveillance and citizen science (Fig. 1). For general surveillance, we note that truly opportunistic reports made by the public are distinct from those made by professionals (land-users) who may have increased awareness through continued professional development training and certainly have a vested interest in the health of crops and amenity planting (Marzano et al. 2015, 2016; Urquhart et al. 2017). For citizen science, we will consider three broad headings: targeted surveys for specific pests and diseases; structured surveys where observers are asked to help at set times and places; and finally citizen science projects that recruit a regular team of tree health volunteers.

### General surveillance

General surveillance, by citizens and land-users, is perhaps the best way for regulatory authorities to increase the early detection of new arrivals (Spence et al. 2019; Environmental Audit Committee 2019; Pawson et al. 2020). By investigating the concerns of observers this approach creates great opportunities to detect the “unknown unknowns” (Morath et al. 2015). General surveillance is widespread in countries across the globe with notable examples including: GB Non‐Native Species Information Portal (GB‐NNSIP), TreeAlert (UK), National Plant Diagnostic Network (NPDN) (USA) (Table 1). The success of general surveillance will be improved by raised awareness of symptomatology and biosecurity in general amongst the public and professional observers, as well as the ease of reporting (Urquhart et al. 2017; Roy et al. 2015). In Great Britain, the TreeAlert reporting tool is a central hub for tree pest and disease reports (case study 1). This is supported by training materials that aim to educate the public providing identification guides. The NPDN is a network of 70 laboratories that fulfils a similar role in the USA, although they have a wider remit covering all plant pests and pathogens in natural and agricultural settings (Table 1).

Encouraging reporting by raising awareness of current outbreaks, as well as species thought likely to arrive or which are high risk, can improve detection by general surveillance programmes. Training materials and programmes may be best when focused toward land managers, as these individuals are most likely to encounter affected trees (Baker et al. 2018; Marzano et al. 2016), although there are also many amateur naturalists who are regularly engaged in reporting sightings of species through organisations like the National Biodiversity Network (NBN) and significant efforts have been made to raise awareness of invasive species through its associated GB‐NNSIP programme (Roy et al. 2015). The production of training materials and direct engagement with observers, begins to move even general surveillance away from being a purely passive process, and the distinctions with citizen science begin to blur.

### Targeted surveys

Targeted citizen science surveys for individual pests and diseases have been successfully deployed many times in order to record the distribution of affected sites or causal agents, examples include: the OPAL (OPen Air Laboratories) tree health survey, which focused on 6 priority pests; the search for spittle bug vectors of *Xylella fastidiosa* as part of the Brigit project (which is investigating the threat posed from this vector borne disease to plants to the UK); and *Phytophthora* species in South Africa (Table 1). Volunteer data collection can also be of use to map the distribution of host plants in advance an invasion, for example, in Denver a programme is actively seeking to document the ash distribution to aid emerald ash borer (*Agrilus planipennis*, EAB) management and raise awareness of its symptoms and impact before this pest arrives (Zentz et al. 2020) (Table 1). These programmes raise awareness of specific pests, provide the information needed to identify potential cases and ask observers to look for occurrences local to them. These are extremely useful as a responsive action to a newly detected or established species where its range is unknown. This targeted approach can yield useful results very quickly. For example, species distributions of native wasps have been successfully estimated following two weeks of data collection by observers (Sumner et al. 2019). In addition to mapping species distributions targeted surveys can also play a vital role in directing management efforts (Forestry Commission 2020).

### Structured and semi-structured surveys

Structured and semi structured surveys provide a great option to reduce sources of reporter bias in citizen science datasets. Observers can be directed to locations and times that are more representative of a random sample. These approaches have recently been applied to the National Plant Monitoring Scheme (NPMS) in the UK, where at the beginning of each sampling season sampling locations are selected using a weighted random sample based on land use and observers are asked to contribute at any of these locations (Pescott et al. 2015, 2019). Using this method observer contributions are encouraged to fit a balanced design and the distribution of surveyed sites can be directly assessed for bias against the background of selected sites. Similar methods could be used for pest detection and would be easily extended through blitz style approaches where training events and therefore subsequent detections occur at predetermined locations (Meentemeyer et al. 2015).

An extension of structured surveys is to integrate active and passive surveillance programmes. This could be achieved either by attempting to direct observers to report in set locations or perhaps more practically by ensuring that active surveillance responds to correct reporter bias. The simplest method of integrating active and passive surveillance is to design a structured survey around a known dataset of volunteered data (Brown et al. 2017a), although if surveys are ongoing there are opportunities for predictive or real time approaches where paid surveyors fill gaps in observer data (Tulloch et al. 2013).

### Tree health volunteers

The most involved form of passive surveillance involves training a regular and committed team of volunteers who can frequently report pest and disease sightings and even contribute by following up on general surveillance enquiries. In Great Britain, the Observatree project focuses on 22 priority pests and diseases and provides identification guides for species identified in horizon scanning exercises (Table 1). In the USA the first detector programme offers a similar set of training materials focusing on identification, surveying and sampling to detect pests and diseases (Table 1). Tree health volunteers are also well placed to integrate with active survey efforts and have contributed to activities beyond the main outbreak zone in the UK (see case study 2).

### Desktop approaches

There are further examples of passive surveillance that have been shown to be extremely useful, but are beyond the remit for inclusion in this review and won't be discussed in full, these are desk based activities which involve remote sensing and mining existing datasets for disease data. The availability of satellite data has made wide scale monitoring of environmental and vegetation change possible (Trumbore et al. 2015). Often the cause of change is not known without follow up field visits to identify causes of the change and confirm the presence of pests and diseases (Mahon et al. 2002), however this may soon change. For crop trees grown in monoculture, pre-symptomatic infection by *Xylella fastidiosa* can already be identified from the air (Zarco-Tejada et al. 2018). Existing biological recording schemes may also already contain useful information regarding many pests and diseases. For example, the UK (and the Netherlands) have light trap networks for monitoring moth populations which can be used to detect pest species (Pocock et al. 2017b). Images stored in online databases may also have vital information about pest and disease distributions, for example photos of flowers stored in iNaturalist can be used to detect anther smut (Kido and Hood 2020) and google streetview has been used to detect pine processionary moth (*Thaumetopoea pityocampa*) in France (Rousselet et al. 2013).

Case study 1: TreeAlert The web-based reporting system TreeAlert was designed as a portal for the general reporting of tree pests and diseases in Great Britain, following an outbreak of chalara ash dieback (*Hymenoscyphus fraxineus*). This new interface was added to encourage easy and rapid reporting of suspected cases to an established tree health diagnostic and advisory service operated by Forest Research. Initially TreeAlert was created only to report this one disease on ash, but it was soon re-developed for reporting of any pest or diseases on any trees in Britain. It is now a valuable aid for forestry and tree professionals as well as members of the public, allowing them to quickly report any suspect findings of tree pests and diseases. TreeAlert is the only online tool in the country created for the purpose of detecting all tree health issues, whether novel or established, and forms part of an early warning system to protect our trees. The effectiveness of TreeAlert depends completely on the reports submitted by users. Apart from the rapid detection of new threats, the reports also give an indication of the most common disorders currently affecting trees, as well as gathering information about the general health of the nation’s trees, woodlands, and forests. The data obtained through TreeAlert is used to enable follow up site visits, to identify trends of spread, to direct surveys looking for regulated or quarantine pests or pathogens and to support pest and disease management.

Initially TreeAlert was developed as a smartphone application to report suspected chalara ash dieback cases and an extremely high number of reports were generated in the first two years. However, the quality of the reports was often insufficient to be followed up by official agencies. In many cases the information uploaded was incomplete or inappropriate, the location was not clearly defined, there were no photographs, or the photographs were not of good quality for an initial triage. As a result, almost every case needed to be followed up either by email or phone, more staff were needed to follow up every case and to do the triage of the reports. This used a significant proportion of the available budget conducting administration tasks and left limited time for rather than doing laboratory diagnostics or research. Re-development of TreeAlert as a purely web-based system aimed to solve these issues. The pages now engage an intelligence system to filter reports and ask more detailed questions where necessary to clarify whether the reporter was reporting specific pests or diseases. Now some data fields are made mandatory, the geographical location is clearly shown on a map and as a grid reference, and three mandatory photographs are requested (to show the tree in context, symptom in context and a close-up of the symptom). The number of reports after re-development initially declined, but the quality of the reports was far higher, for example with the TreeAlert App less than one percent of reports resulted in confirmation of Chalara ash dieback and after re-development as a web-based, 29% resulted in confirmed cases. TreeAlert was extended to report pests and diseases on all trees in 2013 and since the re-development as a web-based tool the number of reports have increased every year. Quarantine pathogens have been reported through the system and, in some cases this has led to eradication (Pérez-Sierra et al. 2019).

Case study 2: Observatree The Observatree project is a multi-partner project in Great Britain that trains and mentors a dedicated group of tree health volunteers. Originally funded by the Department of Environment, Food & Rural Affairs (Defra) as a feasibility project, the project won 50% funding from the EU’s LIFE Programme (the EU’s funding instrument for the environment and climate action) in 2013. Following the success of this earlier stage, the project continues supported solely by Defra and the project partners. The project was designed to create, manage, and support a network of 200 volunteers who receive extensive training in the identification of 22 priority pests and diseases. These observers survey their local woodlands and trees, recording general tree condition and submit reports (via TreeAlert) when any of the 22 priority pests or diseases is suspected to be present. A full range of resources (field guides, posters, videos, and webinars) were created for training purposes and to aid the identification of tree pests and diseases in the field. Each year 12 face to face training events are provided and these are supported by additional networking and mentoring events. The 22 pests and diseases selected comprise a mixture of those already present and others that are not thought to be present but have the potential to have a serious detrimental impact on British forests. By including pests and diseases that are already present to the programme ensures that can make successful detections and most importantly monitor their spread where they have historically been present only in certain parts of the country or were recent arrivals to Britain. For example, the horse chestnut leaf miner (*Cameraria ohridella*) is common in Britain, but sightings are of interest in the north of England and Scotland, where its presence is not well known. A second example can be found with the recording of a recently introduced pathogen, *Sirococcus tsugae* on cedars, where observers not only recorded the positive findings, but they also gathered absence data where they found only healthy trees. Recording absence data is one of the roles of Observatree volunteers and this activity supports active surveillance. Volunteers, scientists and authorities collaborated extensively during the oriental chestnut gall wasp (*Dryocosmus kuriphilus*, OCGW) outbreak in Kent in 2015. The first finding was by an amateur gall enthusiast who reported it to the authorities. As a result of this finding, scientists quickly prepared a webinar to train Observatree volunteers on the identification of this pest. Within a week of the webinar, a second report was submitted via TreeAlert by an Observatree volunteer who detected this pest in Hertfordshire (approximately 100 km away from the initial report). This finding was communicated to the authorities who quickly reassessed the structure of active surveillance for this pest.

In the last 5 years the number of different priority pests and diseases reported by Observatree volunteers has increased from six in 2015 to 11 in 2019. Observatree volunteers also report on the general tree health condition of trees in Britain and have submitted over 10,000 reports in the last 5 years. The majority of these reports relate to healthy trees, providing important baseline data that potentially could allow scientists to monitor rates of spread when new pests or diseases are found in any given area.

One of the potential concerns at the beginning of the project was the number of enquiries submitted by Observatree volunteers would overwhelm the TreeAlert system. This concern has proved unfounded, as Observatree volunteers were asked to only report suspected cases of the priority pests and diseases as well as potential sightings of novel “unknown” pests. Observatree volunteers report any absence or other general tree health data independently to project staff. Observatree enquiries represent around 10% of the total number of TreeAlert enquiries received annually.

The number of individuals reporting priority pests and diseases within the Observatree network has changed every year with only 17 of the volunteers reporting priority pests and diseases via TreeAlert in 2017 and 66 in 2019. Within the individuals submitting TreeAlert reports, there are ‘super-volunteers’ who submit a high number of observations, whereas on average most observers send a handful of TreeAlert reports a year. These volunteers have often chosen to specialize in a specific pest or disease and provide valuable information on its distribution.

Within the Observatree project, the emphasis has always been on observers submitting high quality reports rather than on high numbers. Over time, observers’ confidence and abilities have grown, and this has been reflected in their activities. Since the project began, Observatree observers have, and continue to be, the extra pair of eyes on the ground, dedicating their time to do routine surveys and to report any suspected priority pests and diseases. All the data that they provide, both positive and negative, contribute to a better understanding and knowledge of the health of trees in Britain.

## Understanding and interpreting data from passive surveillance

The continued expansion of passive surveillance schemes in plant health (Pocock et al. 2017a; Brown et al. 2017a; Baker et al. 2018; Meentemeyer et al. 2015; Ryan et al. 2018; Caley et al. 2019; Rallapalli et al. 2015) presents an opportunity for a step change in availability of surveillance data and pest detection capabilities. However, data from passive surveillance are generally “messy” (Dobson et al. 2020) or “noisy” (Isaac and Pocock 2015) and can be difficult to understand. That is, they are usually collected in an ad-hoc or opportunistic manner rather than by a strictly systematic study design (Fig. 1). This limits the ability to make inferences from data and thus to answer key practical questions for pest risk assessment and outbreak response. For example, given a pest is not known to occur in an area, what is the likelihood it really is absent and just not yet reported? If a pest is present, is it increasing in prevalence and what is its current spatial extent? Recent studies have made some progress in our understanding the contribution passive surveillance to address such questions in plant health. For example, Brown et al. (2017b) found that landowner reports of acute oak decline were able to accurately represent the distribution of the disease, as confirmed by randomised nationwide surveys. Pocock et al. (2017a, b) demonstrated that Oak Processionary moth (*Thaumetopoea processionea*) incursions could be detected by moth recorders, and Meentemeyer et al. (2015) used citizen science to accurately predict the spatial extent of sudden oak death in California.

**Fig. 2:**
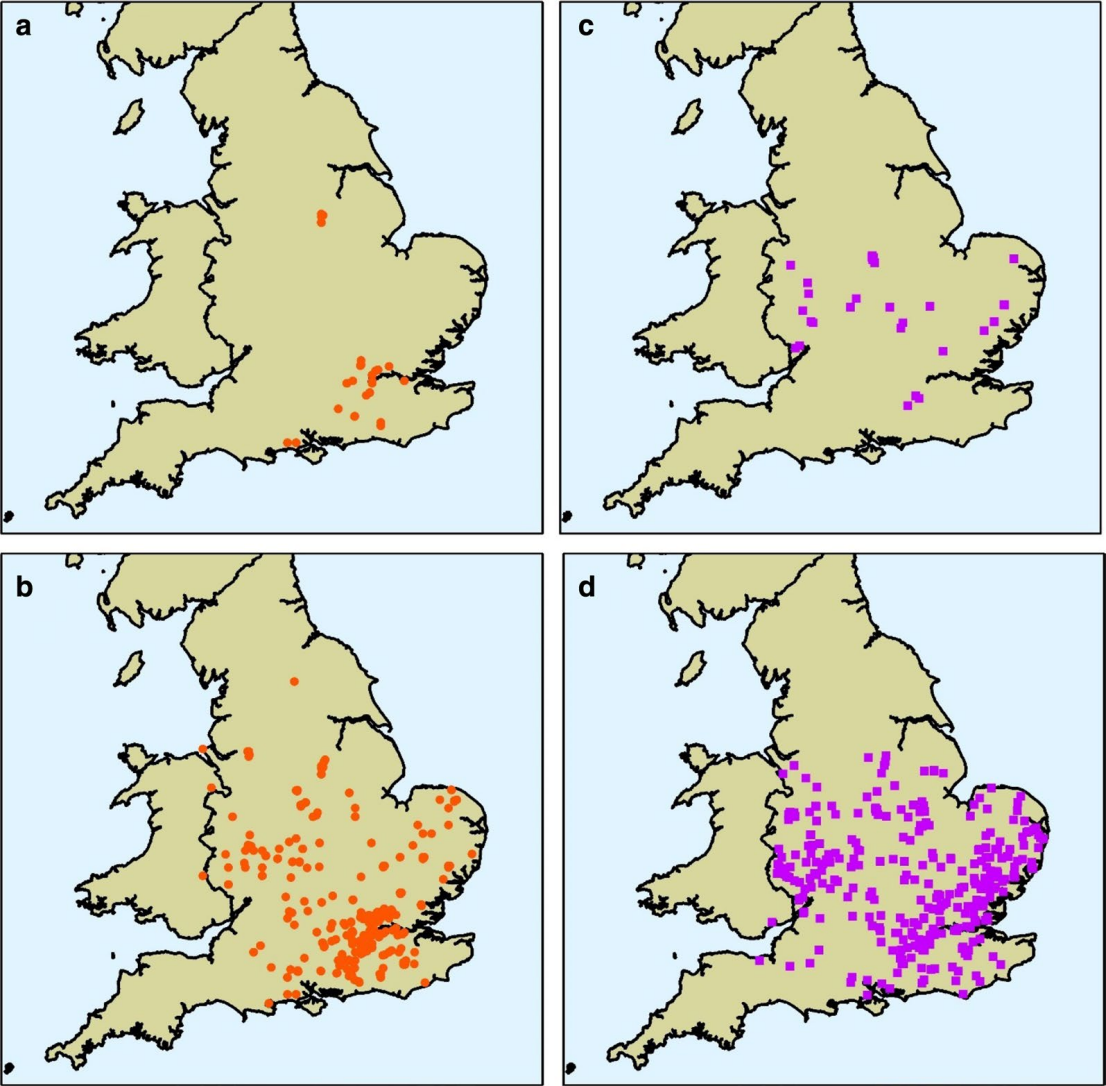
Comparison of reports collected through two passive surveillance programmes, each at two time points. Data are shown for sightings of *Agrilus biguttatus* (a native beetle) collected through the National Biodiversity Network (NBN) and for Acute Oak Decline (AOD, an emerging decline disease where *A. biguttatus* has been implicated) collected by Forest Research (Brown et al. 2017b; Doonan et al. 2020). For both datasets, the current distribution of observations is shown alongside maps containing only earliest historical records. **a** Shows NBN reports before 1987 (when Shirt published a red data book for insects (Shirt 1987)) and **b** shows all NBN reports to 2017; **c** shows Forest Research records before 2009 (when Denman and Webber first described AOD (Denman and Webber 2009)) and **d** shows Forest Research reports up to 2017. Data are discussed further by Baker et al. (2018)

Despite this potential, there are numerous challenges with passive data collection that must be overcome to enable reliable inference for future projects. For example, the distribution of *Agrilus biguttatus* reported through the National Biodiversity network (NBN, Table 1) covers a broadly similar region of Great Britain to reports of Acute Oak Decline submitted through TreeAlert, however they are noticeably different in intensity across this distribution and this was especially true in the past, when only a small number of records were available (Fig. 2). Many of these challenges are not unique to plant health and apply equally well to wider environmental monitoring. Failure to overcome these challenges will undermine the usefulness of data from passive surveillance. Kamp et al. (2016) compared both unstructured citizen science data and structured long-term monitoring data to predict population trends in 105 bird species and found that the former was unable to detect declines in many species. This was due to an inability to account for the numerous sources of bias introduced from unstructured data for example, reporting bias toward certain popular species. In general, the usefulness of passive surveillance data in plant health will increase for structured or semi-structured schemes where sources of bias can be accounted (for example Brown and Williams 2019). However, data from any scheme can be made use of given information on who, when, where and how a record was taken. In the sections below we outline some of the key data challenges facing plant health passive surveillance and identify some approaches that can be used to overcome them.

### Probability to detect and report

A common concern when using data collected by citizen scientists is the ability of members of the public to correctly identify the pest or disease in question. Recent studies have suggested that the sensitivity of detection of invasive species by citizen scientists is, in some cases, comparable to that of experts (Meentemeyer et al. 2015), given suitable training (Gardiner et al. 2012) and retention of observers over time (Dickinson et al. 2010). This will clearly vary depending on the nature of the scheme, with those such as Observatree in the UK (see case study 2) or the USDA First Detector Program (Stubbs et al. 2017) that offer prolonged training expected to achieve higher detection probabilities than those that rely on ad-hoc reports from members of the public (Fig. 1). This suggests that the quality of the data obtained through passive surveillance schemes should not be a constraint to data use.

Detection ability is a combination of an observers ability to correctly identify a pest where it is present (sensitivity) and correctly identify its absence where it is not present (specificity). For data analysis and epidemiological modelling, quantification of an observers sensitivity and specificity at the scale of a predefined inspection unit (i.e. a plant, a woodland, a hectare or any other discrete area containing susceptible host plants that can be inspected or sampled) needs to be available. That is, if the inspection unit is an individual tree, we need to be able to quantify the probability that an observer will detect the pest or disease in an individual tree if it is present. For example, recent epidemiological approaches to estimate the prevalence that an invading pest will have reached once it is first discovered by a surveillance process, rely on accurate estimates of detection probabilities (Parnell et al. 2015; Mastin et al. 2019) as do approaches to ascertain evidence of pest freedom from plant health surveys (EFSA 2019). Detection ability will vary greatly among reporters, with participants varying in skill level from novice to trained amateur pathologist or entomologists, making quantification challenging. This is confounded by variability in the probability to report among recorders, this could be due to lack of willingness to report or adopt the subsequent biosecurity measures or lack of awareness of reporting mechanisms themselves (Urquhart et al. 2017). Moreover, detection probabilities will not only differ depending on the individual recorder, but also on the species of concern. Caley et al. (2019) found that the physical features of insect species had a strong effect on detection and reporting probability, and thus that larger more colourful pests such as *Monochamus* spp. (e.g. which include vectors of pine wood nematode) would have higher reporting rates than smaller vector species such as *Diaphorina citri* (vector of citrus huanglongbing). Seasonality in the life cycle of pests and the expressions of symptoms of plant pathogens will also greatly influence variability in detection and reporting probability.

Quantifying detection probability therefore relies on untangling a range of processes including the probability of pest presence, probability to detect and the probability to report (Hester and Cacho 2017). Ideally, the detection ability of an observer would be quantified by expert validation of observer reports involving confirmation of symptoms via appropriate laboratory and diagnostic methods. This could be used to quantify specificity and sensitivity for individual recorders, but more usefully to quantify variability among observers for different passive surveillance sources and pests. However, expert validation on the scale required may be costly and negate the original benefit of using low cost passive surveillance. For this reason, studies have looked to estimate detection ability indirectly or to include it as a covariate in models. For example, Johnston et al. (2017) used ‘expertise scores’ (a measure associated with how many species an observer reported on average) as a covariate to successfully explain inter-observer variability in detectability in the eBird citizen science dataset.

### Variability in sampling effort and lack of absence data

One major constraint associated with passive surveillance data, compared to systematic surveys, is the lack of ‘negative’ (species absence) data. That is, observers will tend to report when they find a positive occurrence, but not report when they have visited a site, but not made a detection. This is confounded by the fact that citizen science effort is likely heavily biased to certain areas (e.g. residential or frequently visited areas). This makes it difficult to clearly determine the prevalence and spatial extent of the pest (or even the exact population from which the data was collected) (Václavík and Meentemeyer 2009; Isaac et al. 2014). To use a simplistic illustration, if 100 sightings of a pest are recorded by a citizen science scheme in a given period, if 1000 inspection units were visited then prevalence could be estimated at 10%, but if one hundred thousand inspection units had been visited, the estimated prevalence would be closer to 0.1%. Thus, without denominator information (i.e. ‘absence data’ revealed by the number of units inspected), pest record data alone may not be particularly useful (except perhaps to simply confirm presence of a previously absent pest).

In the case of structured or semi-structured passive surveillance schemes, this can be dealt with at the survey design stage e.g. through the allocation of distinct survey units and observation periods. This approach has proved successfully with bird monitoring schemes such as the BTO Breeding Bird Survey (Table 1) where participants are allocated distinct 1 km squares to survey with a specific monitoring protocol. However, where data are collected more ad-hoc or opportunistically this can present more of a challenge. In some cases, it may be possible to use other variables as a proxy for absence data. For example, Pocock et al. (2017b) used routine observations of native moths as a proxy for survey effort for rarer detections of oak processionary moth. Similar use of ‘inferred absences’ have been successfully used to map species from other taxa using citizen science data (for example, Bradter et al. 2018). The issue of lack of absence data is commonly encountered in models of species distributions (for example, Elith and Leathwick 2009). These approaches are commonly used to predict the potential distribution or ecological niche of a pest. This is done by determining the relationship between locations where a pest has been reported with the environmental conditions at that location. The process relies on both presence and absence data, since the latter are required to identify environmental conditions where a pest cannot establish, rather than simply where it has not yet been reported. This can be partially offset by the use of background data or pseudo-absence points, yet presence-absence models are still generally more reliable (Syfert et al. 2013).

Various statistical methods of incorporating presence-only data when developing SDMs have been proposed, including inhomogeneous point process models (Renner et al. 2015; Warton and Shepherd 2010). These allow the explicit modelling of spatial biases in sampling effort, as well as ecological variables influencing the species distribution itself (Warton et al. 2013). If combined with data collected by active surveillance, correlations between observer bias and environmental associations may be able to be accounted for, and an absolute measure of species occurrence (rather than a relative measure) obtained (Fithian et al. 2015; Dorazio 2014; Giraud et al. 2016). Point process models have also been used for the mapping and modelling of the spread of invasive species (Balderama et al. 2012). Occupancy-detection models, which involve repeated visits to the same site to estimate the conditional probability of absence, and also explicitly model the data collection process, have also been shown to accurately estimate species distribution when survey effort is variable and information lacking (for example, Isaac et al. 2014).

### Detecting rare events

Little work has been conducted to date on quantifying the benefits of passive surveillance in early detection surveillance, where the species of interest is generally absent but appropriate actions must be instigated upon discovery. Instead, most studies of citizen-led invasive species detection have focused more on estimating maps of potential suitability of an invader (e.g., César de Sá et al. 2019), documenting the spread of a species post-invasion (Brown et al. 2018) or otherwise determining the extent of spread in cases where control has not been achieved (Pocock and Evans 2014; Meentemeyer et al. 2015).

Early detection presents unique data challenges, including lack of previous encounters with the pest which may result in particularly low detection abilities, compared to experts (Fitzpatrick et al. 2009). However, citizen science still offers much potential for early detection of invasive pests, given the large number of potential observers (Larson et al. 2020; Silvertown 2009). Early detection has been the focus of one farmer-led passive surveillance study which investigated the detection of low-pathogenicity avian influenza (LPAI) in Italian turkey flocks (Comin et al. 2012). This study attempted to reveal the effect of different surveillance strategies on the detection of LPAI, often has mild clinical signs in poultry) within farms. In order to achieve this, a simulation model of pathogen spread was created, which accounted for both within-farm and between-farm spread and both active and passive surveillance within farms (Comin et al. 2011). The overall conclusion from the analysis was that passive and active surveillance in combination worked well for detection, but that passive surveillance was less useful in the absence of clinical signs. This is likely to pose a significant issue for the detection of plant pathogens with long asymptomatic periods, such as *Xylella fastidiosa*, where laboratory testing of asymptomatic tissue or vectors is the only viable method for early detection (Lázaro et al. 2020). However, pathogens with shorter asymptomatic periods and more distinctive symptoms, as well as larger insect pests, could offer excellent candidates for early detection via citizen science. Parnell et al. (2015) developed a framework to estimate the prevalence an invading pest will have reached by the time it is first detected by a surveillance program. By incorporating information on the detection lag of a pest as well the detection sensitivity of the detector, the number of observers needed to achieve early detection of an invading pest population can be determined. Passive surveillance programs where significant number of observers can be recruited may be effective even for pests associated with long detection lags and low detection probabilities.

## How can we design a reporting system to get the most from observer efforts

Many forms of passive surveillance and citizen science reporting are currently available, and many further novel approaches are undoubtedly possible and will arise in the future aided by both technological advances and nimble minds. Given this variety it would be presumptive to give a definitive outline for best practice when designing new schemes, however there are certain that seem to be fundamental to good citizen science and passive surveillance efforts.

A key first challenge when collecting observer data is making sure that there are simple mechanisms for reporting, so that there are limited barriers to participation (Pocock et al. 2014). Passive surveillance records have historically been collated from phone, letter, and email correspondence to advisory bodies (Gilbert et al. 2005; Potter et al. 2011), but this is a labour-intensive process and does not suit uniform data collection or mass participation. Web and app-based reporting schemes have great advantages for streamlined consistent reporting. Ideally the reporting process will ask for the minimum amount of data to accurately locate and identify causal agents (Pocock and Evans 2014). In many respects passive surveillance represents the ideal problem to engage observers, as it works best when there are many participants and there is no requirement for repeated participation (Pocock et al. 2014).

Once a reporting mechanism is in place it needs to be adequately supported, there need to be staff available to check, process, and validate reports so that specific pests and diseases can be reliably identified. Depending on the pest in question, validation may be as simple as using photographs provided by the observer, but often requires field visits and/or processing of laboratory samples. Validation ensures information flowing to plant health officials is accurate and is an important first step towards reliable analysis (Brown et al. 2017a; Steen et al. 2019). Volunteered data can be most readily used when host species are readily identifiable, and symptoms are distinct (Crall et al. 2011). The process of validation is especially important for surveillance datasets where the number of reports may be limited. In certain cases, and with the correct infrastructure, citizen science volunteers can act to self-validate reports. In New Zealand the “Find-A-Pest” programme showed that observers validated 99% of reports (only 1% remained unconfirmed when passed to plant health professionals) with 95.5% accuracy (Pawson et al. 2020).

The above simple rules apply to any form of passive surveillance, but further methods can be applied to improve the various forms of reporting. The analysis of volunteered geographical data is simplified if datasets also contain absence data (symptom free locations are also reported), however this is difficult to include as part of a general surveillance programme where the scope of reporting is broad in focus and not targeted at specific pests and/or symptoms. The wide-ranging nature of general surveillance data makes it especially useful for first detections of new introductions, but the scope for using these reports is not limited to this function. Presence only data can be included in analyses; it just becomes especially important to assess the datasets for spatial bias, which can be achieved by investigating the distribution of a specific pest or disease against the background of all reports received. For example, the probability of detection for Oak Processionary moths was estimated across Great Britain using the distribution and activity levels of individuals in an established light trapping network (Pocock et al. 2017b). A similar approach has recently been used to assess bias and improve species distribution models for 138 bird species across Great Britain (Johnston et al. 2020). Both examples show the benefit of having large diverse datasets that contain multiple species and regular observers who report many different species. A general surveillance programme therefore benefits from high volumes of reports and from collecting information across a wide range of issues. Even established and widely occurring issues can be of great value, as they have the potential to reveal valuable details about reporter behaviour.

In addition to making comparisons within a dataset, there may be additional benefits to conducting analyses across datasets, as reporter behaviour may differ between programmes and accuracy could be improved through comparative analysis that “triangulates” predictions (Dobson et al. 2020; Baker et al. 2018). Such analyses are uncommon in ecology and disease surveillance, but comparable datasets are increasingly available, and networks are being developed to foster links between programs. For example, the UK has established a tree health citizen science network and more formally New Zealand has established the government industry agreement for biosecurity readiness and response (GIA) (GIA 2020). A key component to designing a new monitoring scheme should be to make an assessment of what partnerships can be formed with existing volunteer groups and professional bodies.

There are clear benefits of large-scale, integrated working in passive survielance programmes, however there remains a vital place for individual targeted citizen science surveys, as they provide a fast way to respond to new discoveries and developing situations. When a pest or disease is first discovered it becomes crucial to understand how well established it is and while active surveillance will be directed towards the outbreak zone passive surveillance can contribute greatly to the surveillance of the wider landscape. If targeted surveys are implemented there will be great benefits to capturing absence data in addition to any new detections, observers should be encouraged to go and look for symptoms and submit their findings regardless of the outcome of their search. A good way to achieve this, while also gaining additional information about population size or prevalence is to incorporate a trapping or sampling element into the survey design. For example, the conker tree science project asked participants to collect leaves place them in a sealed bag and count horse chestnut leaf miner (*Cameraria ohridella*) emergence numbers (Pocock and Evans 2014) and the tree body guards project asked school children to make plasticine models of caterpillars and place them on oak trees, so that researchers could count marks made by predators (Castagneyrol et al. 2020) (Table 1).

The benefits of volunteering are well defined (O’Brien et al. 2010), but understanding what motivates observers and how to maintain the engagement of specific groups of observer is key for the success of the activity or project—this is especially true for more complicated systematic surveillance approaches that require repeated reports across large geographical areas (Pocock et al. 2014). Observer motivation could be due to a diverse set of reasons, for example: volunteers may act through: personal interest and enthusiasm about the activity; care for the environment; feeling useful; ‘doing the right thing’, or a sense of duty; or make a contribution to the community. As such, keeping observers engaged over time can be challenging and although engagement needs to be tailored to the specific observer groups, there are some general concepts that might help keeping observers engaged. It is important the collective results are reported back frequently, so observers can see how they fit in the bigger picture. Ensuring projects have clear goals and objectives simplifies this process (Ambrose-Oji 2011). It is important to maintain continuous communication and feedback, especially to make sure contributions are acknowledged and to establish and manage expectations at the outset. Communication can take place individually, but also through a variety of media, newsletters, social media, as well as, at training and mentoring events (Ambrose-Oji et al. 2014). Finally, it is essential to provide the right tools for the activity they are asked to do (O'Brien 2015) and understand that not every person learns in the same way and so be prepared to offer a range of different approaches. Focusing on understanding bias within reporter activity can be informative in relation to understanding their motivations and help to tailor future messaging and goals to fit the profile of established groups (Boakes et al. 2016). Conversely, efforts to diversify the pool of observers, who are often older, well-educated, more rural and more well off than individuals who do not take part in biological recording programmes (Mac Domhnaill et al. 2020) and make programmes more inclusive could lead to larger number of reports, as well as, potentially reducing bias.

## Conclusions

Passive surveillance already is playing a crucial role in the detection of plant health issues across the globe, however, approaches to gather and work with this data are far from universal (Dobson et al. 2020). Research can contribute greatly to inform the standardisation of methods and to improve data analysis. Where observers are numerous and providing repeat observations methods from species distribution modelling can be applied to understand bias and improve model accuracy (August et al. 2020; Isaac et al. 2014; Renner et al. 2015). For plant health issues, it remains particularly important to understand the components that affect detection (namely the probability to detect, probability to identify and probability to report the issue), so that inferences can be made quickly when new threats are discovered.

The examples presented in this review are drawn mostly from the field of tree health, but passive surveillance also has the potential to contribute greatly in agricultural (Wright et al. 2018; Mutembesa et al. 2018), as well as, animal and human systems (for example Covid19, Table 1) especially where information is needed quickly or in real time. There are many options available for groups wanting to develop or expand passive surveillance or citizen science reporting (including great potential for the development of novel ideas), what we believe is crucial to their success is the consideration of how data are to be used before collection begins. The goals and target species for passive surveillance must be carefully chosen for a programme to be successful, there is no point in setting impossible challenges. With appropriate training volunteers can excel at detecting invasive species, however high-quality training materials alone will not guarantee success. In the right circumstances (easily identifiable pests or symptoms and abundant or obvious host species) a mass engagement approach can quickly yield large scale data, however if more complex questions are to be addressed or ongoing monitoring is needed then other approaches (such as a tree health volunteer programme) may be required. Across the spectrum of passive surveillance, options are already available to address many of the challenges facing plant health authorities and the suite of options for analysts to interpret such data are growing rapidly.

## Data Availability

Not applicable.
